# Anesthetic management for cesarean delivery in a pregnant woman with polymyositis: A case report and review of literature

**DOI:** 10.1186/1757-1626-2-9107

**Published:** 2009-11-29

**Authors:** Ilkben Gunusen, Semra Karaman, Seymen Nemli, Vicdan Firat

**Affiliations:** 1 Department of Anesthesiology and Reanimation, Ege University Faculty of Medicine, Izmir, Turkey

## Abstract

**Introduction:**

Polymyositis which is a rare disease both in general population and in pregnancy is systemic connective tissue disorder characterized by inflammation and degeneration of muscles. There is only a little information relating to the anesthetic management of a pregnant woman with polymyositis.

**Case presentation:**

In this article, we present anesthetic management of urgent cesarean delivery of a 28-year-old parturient with polymyositis under epidural anesthesia who was diagnosed with polymyositis five years ago and has been treated regularly with different doses prednisolone since then.

**Conclusion:**

In a parturient with polymyositis, it should not be suggested general anesthesia due to risks including delayed recovery from muscle relaxation, aspiration pneumonitis, arrhythmias, cardiac failure, we consider that epidural anesthesia for cesarean section can be safely applied.

## Introduction

Polymyositis (PM) with the prevalence from 2.4-10.7 cases per 100.000 persons in the general population is characterized by symmetric proximal muscle weakness, increased serum skeletal muscle enzymes, electromyography (EMG) abnormalities and inflammatory cell infiltrates in muscle tissue. Hormonal factors, environmental exposures and genetic factors are suggested to contribute to the onset of this disease with unknown etiology [1,2]. While symmetrical, bilateral neck, shoulder, and pelvic muscle weakness are seen in 50% of these patients; intercostal and diaphragmatic muscles and pharyngeal muscles may also be affected. Although corticosteroids are the mainstay of treatment, unresponsive patients or patients who develop undesirable side effects are given immunosuppressant (azathioprine, cyclosporine, methotrexate) or immunoglobulin [3,4]. We present our case report on the grounds that there are only a few reports available in literature on the anesthetic management of polymyositis in pregnant women [5,6]. Furthermore, in the case reports on the operations of other patients who underwent regional anesthesia, there was not enough information about clinical course and muscle functions in postoperative period.

## Case Presentation

A 28-year-old, white, 158 cm tall, 68 kg primigravida woman at 38 weeks' gestation was referred to our hospital as her labor pain had started. She was diagnosed with polymyositis by muscle biopsy and EMG five years ago, and the dose of prednisolone was readjusted due to muscle weakness in pregnancy. For the last month of the pregnancy, the dose of oral prednisolone has been inceased from 5 mg to 30 mg once daily.

No family history of polymyositis or any muscular disorder was present. Preoperative physical examination was normal. She had no history of respiratory and cardiac problem. She was a non-smoker and no-drink alcohol. Biochemistry results including liver enzymes (AST, aspartate aminotransferase: 252 U/L, normal limits: ≤ 35 U/L; ALT, alanine aminotransferase: 201 U/L, normal limits: ≤ 40 U/L; LDH, lactate dehydrogenase: 1478 U/L, normal limits: 240-480 U/L) and CK levels (creatine kinase: 1886 U/L, normal limits: ≤ 145 U/L) were high. Neurologic examination on admission showed that muscle strength was 4 (on a 0-5 Medical Research Council scale) for the upper limb proximal muscles, 3 or 4 for the lower limb proximal muscles, 4 for the hip flexion and 3 for the neck flexion. She was found to have a cervical dilatation of 4 cm after the examination and thus the neurologist together with the obstetrician decided on urgent cesarean section owing to her muscles weakness which might lead to difficulties in vaginal delivery.

After written informed consent approval, she was premedicated with metoclopramide (10 mg, i.v.) and ranitidine (50 mg, i.v). Lactated Ringer's solution was started a rate of 15 to 20 mL kg^-1 ^per hour within 15-20 min before epidural anesthesia. In the operating room, electrocardiograph, oxygen saturation via pulse oximetry (SpO2) and noninvasive blood pressure were monitored (Datex-Ohmeda AS/3 Helsinki, Finland). An epidural catheter was inserted at the L4-5 interspace through an 18-gauge Tuohy needle using loss of resistance to saline technique in the lateral decubitus position and placed in 4 cm deep. After epidural catheter placement, the patient was placed supine on the operating table, with left uterine displacement. Lateral uterine displacement until surgical incision was achieved by tilting the operating table to 15°left. Supplemental oxygen (4 L/min) by facemask was given until delivery. A test dose of 2 mL of 2% lidocaine with 1:200000 epinephrine was given through the epidural catheter. After verifying that there were no signs of intravascular or intrathecal placement, epidural anesthesia was induced with 16 mL of levobupivacaine 0.5% and 2 mL fentanyl (100 μg), given in incremental doses of 5 mL every two to three minutes. Sensory block height was evaluated by bilateral pinprick test at the midclavicular line every two minutes. The operation started when sensorial block level reached T4-5 at 11 minutes after anesthesia induction. The time from surgical incision to delivery was 8 minutes, and the patient delivered a full-term healthy baby (2520 g, 49 cm), with 5- and 10-minute Apgar scores of 8 and 10, respectively. After delivery, we administered 10 U oxytocin in 500 mL crystalloid solution by slow intravenous infusion. The patient was hemodynamically stable and showed no signs of hypotension or bradycardia throughout the operation. In the postpartum period, our patient was monitored with a three-lead electrocardiogram (ECG), an automated, non-invasive, Datex Cardiocap blood pressure device, a pulse oximeter and a urinary catheter. She was given morphine (two mg) through the epidural catheter for postoperative analgesia and then the catheter was removed after 12 hours. In the postoperative period, the neurologist again evaluated the patient using MRC (Medical Research Council scale) after the epidural block regressed over a period of 115 minutes. Twenty-four hours later, the patient's new EMG was compared with the previous ones. Her muscle function showed no deterioration from the antepartum period. Our patient was safely discharged home on the fifth postoperative day. Two weeks later, the serum level of AST was 113 U/L, the serum level of ALT was 108 U/L, the serum level of LDH was 940 U/L and the serum level of CK was 884 U/L. No improvement in muscle functions was recorded in the patient's controls after one and six months.

## Discussion

Polymyositis is not common in pregnancy; however, the possibility of development or exacerbation increases when maternal hormonal changes occur. If the disease is not in remission, it should be considered that both the mother and the baby are under high-risk during pregnancy [7,8].

Points to be taken into consideration in the anesthetic management are: respiratory insufficiency, aspiration pneumonia, arrhythmias, cardiac failure and hyperkalemia [4]. It's believed that the patients with polymyositis are sensitive to nondepolarising muscle relaxants, and the use of their antagonist drugs may cause muscle weakness and severe dysrrythmias [9]. Steroids induced myopathy leads to an increased sensitivity to neuromuscular blocking drugs and an unpredictable response may be seen. Doses usually have to be reduced upon titrating against response. Furthermore, volatile anesthetic agents may not only serve as a trigger of malignant hyperthermia but also potentiate the effects of muscle relaxant. It is recommended that these agents should be avoided in patients with raised plasma creatine phosphokinase (CPK) levels. Succinylcholine is also advised to be avoided as it may serve as a trigger for malignant hyperthermia and lead to hyperkalemia [4]. In addition, vecuronium and pancuronium are associated with prolonged neuromuscular paralysis [3,10]. Neuromuscular blockade monitorization using a peripheral nerve stimulator is suggested due to lack of standard recommendations regarding to the application of nondepolarizing muscle relaxants [4,10,11]. There are some publications reporting that particularly atracurium could be implemented as a safe drug under neuromuscular monitoring [11,12]. However, some researchers report that for endotracheal intubation or for maintaining anesthesia, it would be appropriate to apply regional techniques alone or combined with sedation, or not to use muscle relaxant at all owing to limited sources in the literature [13-15]. Fujita et al [15] performed thoracic epidural block, and no problems occurred during and after the surgery so they showed epidural anesthesia to be a successful method. Ohta et al [14] reported anesthetic management of two patients suffering from polymyositis, in one of which no muscle relaxation was used for endotracheal intubation and maintenance of anesthesia. The other patient was operated under epidural anesthesia with sedation.

Safe, successful administration of regional anesthesia in pregnant women requires an understanding of the normal physiologic changes of pregnancy. At term, obstruction of the inferior vena cava by the enlarging uterus distends the epidural venous plexus and increases epidural blood volume. These effects cause a decrease in spinal cerebrospinal fluid volume (CSF) and enhance the cephalad spread of local anesthetic solutions during spinal and epidural anesthesia. CSF volume inversely correlates with level of anesthesia so decreasing CSF volume is associated with higher blocks. In addition, Functional residual capacity (FRC) begins to decrease by the fifth months of pregnancy. This is caused by elevation of the relaxed diaphragm, which occurs as the enlarging uterus enters the abdominal cavity. Both vital capacity and closing capacity are minimally affected but FRC decreases up to 20% at term. The parturient is at greater risk than the nonpregnant patient for developing airway closure and hypoxemia during major regional anesthesia [16]. Regional anesthesia can impair respiratory function by paralysis of the intercostal muscles due to a high block although clinically significant alterations in pulmonary physiology are usually minimal with regional anesthesia. Satisfactory regional anesthesia for cesarean delivery requires a block level to at least the T5 dermatome and this can alter respiratory performance [17]. Spinal anesthesia is an appropriate choice for urgent cesarean sections. There is only one case report on spinal anesthesia in pregnant women with polymyositis in Medline [5]. One of the major disadvantages of spinal anesthesia is higher incidence of hypotension. Although the rapid onset of spinal anesthesia is often advantageous, the rapid onset of sympathetic blockade may result in abrupt, severe hypotension. Hypotension following spinal anesthesia in cesarean delivery is a very common complication, with reported incidences varying from 50% to 100% [18-20]. Thus, epidural anesthesia may be a preference when physicians want to minimize the likelihood of maternal hypotension or when intense motor blockade of the thoracoabdominal segments is not desired. Another advantage of epidural anesthesia is postoperative pain relief [20]. We could prefer spinal anesthesia in this parturient for urgent cesarean section. However, we avoided high block levels which effect respiratory muscles thus chose epidural anesthesia as we could control block levels at ease. Furthermore we wanted to minimize the likelihood of maternal hypotension and bradycardia and to achieve postoperative pain relief. Besides, we needn't have hurried since there was no sign of fetal distress.

Technique of combined spinal-epidural (CSE) might especially benefit this patient. For cesarean section, it combines the benefit of rapid, reliable, intense blockade of spinal anesthesia together with the flexibility of an epidural catheter. The catheter also allows supplementation of anesthesia and can be used for postoperative analgesia. In CSE applications, the spinal and epidural needles may be placed at different interspaces, but most clinicians use the same interspace as we did. However, when the same interspace is used, epidurally administered drugs should be administered and titrated carefully because the dural hole created by the spinal needle increases the flux of epidural drugs into cerebrospinal fluid and enhance their effects [19]. In our case, owing to unpredictable high blocks caused by these effects, we avoided CSE application.

In the light of the information above, we refrained from general anesthesia in our patient with high CPK level, who was under treatment with corticosteroid for a long time, due to the risk of aspiration and possible harmful results of muscle relaxants and the use of antagonist drugs. Our main concern during the anesthetic management of our case was that there were only a few case reports in literature on anesthesia of pregnant women with polymyositis [5,6].

## Conclusion

As far as delayed recovery from muscle relaxation, aspiration pneumonitis, arrhythmias, cardiac failure and steroid supplementation with its complications are concerned as the major concerns for an anesthesiologist; we consider that epidural anesthesia for cesarean section in a pregnant woman with polymyositis can be safely applied.

## Abbreviations

PM: polymyositis; EMG: electromyography; AST: aspartate aminotransferase; ALT: alanine aminotransferase; LDH: lactate dehydrogenase; CK: creatine kinase; ECG: electrocardiogram; CPK: creatine phosphokinase; CSF: cerebrospinal fluid volume; FRC: functional residual capacity; CSE: combined spinal-epidural.

## Consent

Written informed consent was obtained from the patient for publication of this case report. A copy of the written consent is available for review by the Editor-in-Chief of this journal.

## Competing interests

The authors declare that they have no competing interests.

## Authors' contributions

IG and SN in the patient's management. IG is the corresponding author. IG, SK and VF collected data and drafted the manuscript. All authors read and approved the final manuscript.
